# A GMR enzymatic assay for quantifying nuclease and peptidase activity

**DOI:** 10.3389/fbioe.2024.1363186

**Published:** 2024-03-13

**Authors:** Michael Sveiven, Ana K. Serrano, Joshua Rosenberg, Douglas J. Conrad, Drew A. Hall, Anthony J. O’Donoghue

**Affiliations:** ^1^ Department of Bioengineering, University of California, San Diego, La Jolla, CA, United States; ^2^ School of Biological Sciences, University of California, San Diego, La Jolla, CA, United States; ^3^ Department of Electrical and Computer Engineering, University of California, San Diego, La Jolla, CA, United States; ^4^ Department of Medicine, University of California, San Diego, La Jolla, CA, United States; ^5^ Skaggs School of Pharmacy and Pharmaceutical Sciences, University of California, San Diego, La Jolla, CA, United States

**Keywords:** disease monitoring, point-of-care testing, DNA substrate, peptide substrate, enzymatic activity, giant magnetoresistive sensor, GMR

## Abstract

Hydrolytic enzymes play crucial roles in cellular processes, and dysregulation of their activities is implicated in various physiological and pathological conditions. These enzymes cleave substrates such as peptide bonds, phosphodiester bonds, glycosidic bonds, and other esters. Detecting aberrant hydrolase activity is vital for understanding disease mechanisms and developing targeted therapeutic interventions. This study introduces a novel approach to measuring hydrolase activity using giant magnetoresistive (GMR) spin valve sensors. These sensors change resistance in response to magnetic fields, and here, they are functionalized with specific substrates for hydrolases conjugated to magnetic nanoparticles (MNPs). When a hydrolase cleaves its substrate, the tethered magnetic nanoparticle detaches, causing a measurable shift in the sensor’s resistance. This design translates hydrolase activity into a real-time, activity-dependent signal. The assay is simple, rapid, and requires no washing steps, making it ideal for point-of-care settings. Unlike fluorescent methods, it avoids issues like autofluorescence and photobleaching, broadening its applicability to diverse biofluids. Furthermore, the sensor array contains 80 individually addressable sensors, allowing for the simultaneous measurement of multiple hydrolases in a single reaction. The versatility of this method is demonstrated with substrates for nucleases, Bcu I and DNase I, and the peptidase, human neutrophil elastase. To demonstrate a clinical application, we show that neutrophil elastase in sputum from cystic fibrosis patients hydrolyze the peptide-GMR substrate, and the cleavage rate strongly correlates with a traditional fluorogenic substrate. This innovative assay addresses challenges associated with traditional enzyme measurement techniques, providing a promising tool for real-time quantification of hydrolase activities in diverse biological contexts.

## Introduction

Enzymes play a pivotal role in various cellular processes, and their activities are tightly regulated to maintain cellular homeostasis. Monitoring enzymes is essential to understand cellular processes and disease mechanisms, and many enzyme-based disease biomarkers have been identified. For example, serum amylase and serum lipase assays are used to diagnose acute pancreatitis (Walkowska et al., 2022), alkaline phosphatase activity in blood is a marker for liver or bone conditions (Cannalire et al., 2023), creatine kinase tests diagnose Duchenne’s Muscle Dystrophy in neonates (de Freitas Nakata et al., 2021), and elevated lactate dehydrogenase in blood is associated with poor prognosis in COVID-19 patients, potentially for use as a triage mechanism (Martha et al., 2022). Among enzymes, hydrolases are crucial in regulating intracellular pathways and, as a result, influence diverse physiological and pathological conditions. Hydrolases break down macromolecules such as lipids, carbohydrates, proteins, and nucleic acids. In humans, these enzymes are essential for food digestion, wound healing, cell signaling, and immune defense (Riise et al., 2019; Chen et al., 2020; Rack et al., 2020; Salhi et al., 2021). When these enzymes become dysfunctional, they become drivers of cancer, neurodegeneration, autoimmune disease, and heart disease (Liu et al., 2016; Mondanelli et al., 2019; Moll et al., 2020; Sama et al., 2020). Therefore, developing techniques to detect aberrant hydrolase activity as a disease biomarker provides an important tool for clinicians and researchers.

Nucleases are hydrolases that cleave the phosphodiester bonds between nucleotides in DNA and RNA (Garcia Gonzalez and Hernandez, 2022) and can be broadly divided into DNases and RNases based on their ability to cleave DNA or RNA, respectively (Santa et al., 2021). Nucleases hold great potential as biomarkers for many cancers (Balian and Hernandez, 2021). For example, high DNase I activity is observed in patients with oral and breast cancers, while FEN I is a nuclease that is over-expressed in lung, prostate, brain, gastric, pancreatic, and breast cancer. In addition, RNase I is linked to pancreatic cancer (Lauková et al., 2020; Balian and Hernandez, 2021). Peptidases (or proteases) are hydrolases that cleave the peptide bonds between amino acids in a protein chain. Peptidases are involved in all biological processes, including food digestion, blood clotting, and immune defense (Armstrong, 2001; Walsh and Ahmad, 2002; Shpacovitch et al., 2008; Kårlund et al., 2021). Many diseases are characterized by dysfunctional peptidase activity, including cancer, arthritis, and Alzheimer’s disease (Eatemadi et al., 2017; Lucena and McDougall, 2021; Lichtenthaler et al., 2022). Considering the vast influence of peptidases and nucleases on human health, measuring their activities in biofluids is of great interest.

Common nuclease measurement techniques include hybridization assays, immunohistochemistry, reverse-transcription polymerase chain reaction (PCR), enzyme-linked immunosorbent assay (ELISA), mass spectrometry, and western blots (Balian and Hernandez, 2021). Hybridization assays are qualitative and unsuitable for point-of-care testing (Singh et al., 2008), while immunohistochemistry assays are qualitative and require intensive processing (Wang et al., 2014). Reverse transcription-PCR is semi-quantitative and is usually a measurement of nuclease expression, not activity (Wang et al., 2014). ELISA uses fluorophores or chromogenic substrates that measure only enzyme concentration and can have photobleaching issues or incompatibility problems with sample matrices (Zhang et al., 2016). Many of these assays require a microplate reader to measure absorption or fluorescence (Lauková et al., 2020; Balian and Hernandez, 2021). While effective, they are limited by the size and complexity of the spectrophotometers, confining the assays to centralized labs.

Peptidases are typically quantified by fluorescent or colorimetric assays, where cleavage of a peptide sequence leads to a time-dependent increase in signal (Zhang, 2004; Hao Ong and Yang, 2017; Wei et al., 2019). These enzyme assays are amenable to microplate format and have been used extensively for high-throughput screening but have limitations regarding sensitivity, specificity, and adaptability (Oishi et al., 2008; Nozeret et al., 2019; Hammond and Ferro, 2023). Moreover, a persistent challenge inherent to these assays is their susceptibility to background signals, originating from non-specific interactions, autofluorescence, and photobleaching. This susceptibility can curtail the sensitivity and specificity of the measurements, often necessitating rigorous background correction procedures. Several surface-based (*i.e.*, heterogeneous) peptidase assays have been developed that utilize electrochemical, surface plasmon resonance (SPR), or surface-enhanced Raman spectroscopy (SERS) detection. Electrochemical assays are sensitive and amenable to point-of-care use but susceptible to sample matrix effects (Chen et al., 2015; Menon et al., 2020). SPR sensors are label-free and real-time, but concerns exist over the complexity of the optical equipment required (Wang et al., 2019; Chang, 2021). SERS sensors have issues generating a reproducible colorimetric response, which means that the secondary enzyme will need specific conditions for activity (Ding and Yang, 2014). Overall, the assays described exhibit many desirable characteristics, but there is a need for new assays that overcome their shortcomings, especially regarding ease-of-use concerns and equipment complexity.

**FIGURE 1:**
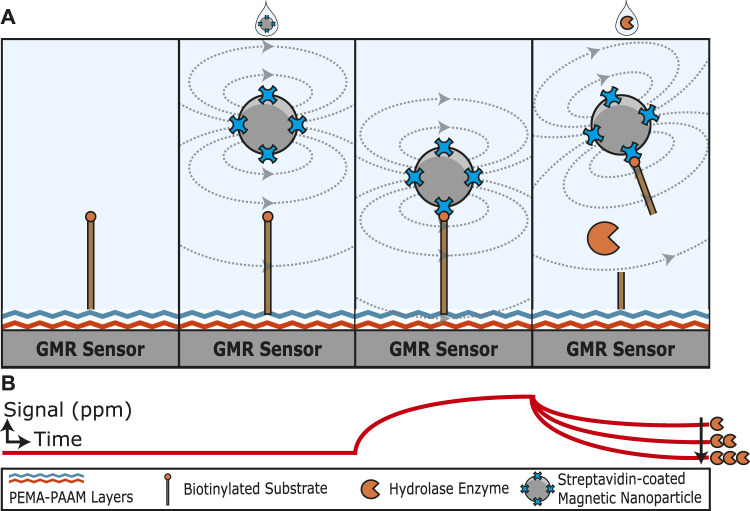
Graphical illustration of a hydrolase assay that uses magnetoresistance to quantify substrate cleavage. **(A)** A biotinylated substrate is covalently attached to the sensor surface through amine coupling. The addition of streptavidin-coated magnetic nanoparticles increases the magnetoresistance signal as the nanoparticles are tethered to the biotin substrate close to the sensor surface. The substrate is cleaved by a hydrolase enzyme and releases the magnetic nanoparticle. **(B)** Illustration of how the magnetoresistance signal changes with time. In the absence of magnetic nanoparticles, a low signal is detected. Upon the addition of streptavidin-coated magnetic nanoparticles, the signal increases with time as the particles bind to the substrate and are orientated close to the GMR sensor surface. The signal then decreases in proportion to the concentration of active hydrolase enzyme added.

This study reports on a new technique that measures real-time hydrolase activity using giant magnetoresistive (GMR) sensors, as illustrated in Figure 1. GMR sensors are elaborately engineered thin-film stacks where the operation is deeply rooted in quantum mechanics; specifically, they exhibit a phenomenon known as spin-dependent scattering. This property makes them very sensitive to changes in the local magnetic field, enabling them to be used as ultrasensitive biosensors (Baselt et al., 1998; Wu et al., 2022). Past research has shown the utility of GMR sensors for measuring antigen levels using an antibody-antigen-antibody sandwich assay. The capture antibody is coupled to the GMR sensor and binds to the antigen. The bound antigen is then quantified using a biotinylated detection antibody as it recruits streptavidin-coated magnetic nanoparticles (MNP) close to the sensor surface (Osterfeld et al., 2008; Gaster et al., 2013; Kim et al., 2013; Klein et al., 2019; Lin et al., 2019; Zhou et al., 2019; Antarnusa et al., 2022; Mostufa et al., 2023; Sveiven et al., 2023). The increase in magnetoresistance directly correlates with the concentration of antigen. Miniaturization of the GMR system has been demonstrated, which allows for greater portability (Olazarra et al., 2022; Gaster et al., 2011; Yao et al., 2022). In the hydrolase assay described here, MNPs are tethered to the sensor substrate via a substrate sequence cleavable by the target enzyme. Therefore, the decrease in magneto resistance over time directly correlates with the enzyme concentration.

A previous attempt to develop a GMR-based peptidase assay utilized a thioether linkage between a cysteine residue on the substrate and a maleimide-coated surface (Adem et al., 2020). However, the thioether bond was insufficiently stable in aqueous solutions and, therefore, not useful for point-of-care assays that require storage of the assembled substrate on the GMR sensor. We have recently functionalized GMR sensors for covalent binding to antibodies via primary amines and, therefore, used this approach to attach hydrolase substrates to the sensor surface. Prior to the addition of the hydrolase enzyme, the signal for the bound substrate can be quantified using streptavidin-coated MNPs. The signal decreases when the MNPs are released following substrate cleavage, enabling real-time, activity-dependent enzyme monitoring. The substrates consist of an amino acid sequence or a nucleic acid sequence to quantify the activity of a peptidase or nuclease. These substrates are flanked by a biotin molecule to capture streptavidin-coated MNPs and an amine chemical group to bind to the sensor surface. GMR sensors allow for a fast, kinetic, wash-free, portable assay with point-of-care capability. As this assay does not involve optical readout, the common issues of autofluorescence and photobleaching are avoided, enabling higher sensitivity. The GMR sensors are arrayed into 80 individually addressable sensors, creating multiplex ability. This study shows the viability of GMR sensors for quantifying hydrolase activity using specific substrates and highlights the potential of this technology to provide physicians and patients with greater opportunities to detect and monitor diseases.

## Materials and methods

### GMR sensor arrays

GMR sensor arrays were purchased from MagArray, Inc (#BZ0078). Each GMR sensor array has 80 sensors arranged in an 8 × 10 matrix, where each sensor is 120 × 120 μm^2^ on a 280 µm pitch with a nominal resistance (*R*_0_) of 1.464 kΩ and a mean magnetoresistance (MR) ratio of 7.99% (Supplementary Figure S1). Each of the 80 sensors can be independently addressed. A custom holder was fabricated from Teflon to create a 100 µL reaction well with an o-ring atop the sensor array (Supplementary Figure S2).

### GMR readout station

The measurement setup consists of a computer, a power amplifier (Texas Instruments, OPA549), a Helmholtz coil (180 turns of 22 gauge wire per coil, resulting in a 40.5 Oe/A coil constant), and custom readout electronics (Hall et al., 2010a), as shown in Supplementary Figure S3. A double modulation readout scheme rejects 1/*f* noise from the sensors and electronics, and a temperature compensation technique is used to reduce the temperature drift (Hall et al., 2010b). The computer digitally adjusts the frequency and amplitude of the sensor bias voltage and magnetic field through a National Instruments data acquisition card (PCIe-6351) and a LabVIEW graphical user interface. Specifically, the power amplifier controlled by the computer provides a current to the Helmholtz coil, which creates a homogenous magnetic field (23–34 Oe_rms_ based on the sensor MR) for the sensor array. The readout electronics contain 8× transimpedance amplifiers to convert the currents to voltages that the data acquisition card quantizes. Time-multiplexing is applied to read the 8 × 10 sensor array with a 10 s update rate. The measured signal is the change in MR from the initial MR in parts-per-million (ppm).

### Surface functionalization

To functionalize the sensor surface, a thorough cleaning process is initiated by sequential washes with 600 µL of acetone, methanol, and isopropanol. Subsequently, the sensor arrays are subjected to a 10-min treatment in an ultraviolet-ozone cleaner (Uvotech Systems, Helios 500). An optimized protocol consisted of adding 100 µL of 1% Poly (allylamine) in 200 mM MES, pH 6.0 to the sensor wells for 10 min, followed by a rinse with 600 µL of deionized (DI) water. The sensors are then baked at 110°C for 90 min in an oven under atmospheric conditions (Thermo Scientific #PR305225G). Poly (ethylene-alt-maleic anhydride) (PEMA) is rendered aqueous by immersing the microcentrifuge tube containing PEMA in a gently boiling water bath (∼100°C) on a hot plate set to 170°C for 90 min, before adding it to the sensor surface. Then 100 µL of 2% aqueous PEMA in 200 mM MES pH 6.0, filtered through a 0.22 µm filter, is placed onto the sensors for 5 min. The sensors are rinsed with 1 mL DI water and then baked at 160°C for 1 h before spotting. This protocol is adapted from a previous study (Kim et al., 2013).

### Reagent spotting

Peptide, DNA, and PEG substrates are diluted in a printing buffer consisting of PBS, 1 M betaine, and 12.5% 2,3-butanediol. Individual sensors are spotted with the substrate using an iTWO-300P automated spotter (axiVEND, Florida). Twenty droplets of ∼100 pL are spotted on each sensor, sufficient to cover the sensor (Supplementary Figure S4). The automated spotter chamber is then brought to 70% humidity for 1 h, and the sensors are incubated overnight.

### Stability assay

To assess the stability of the functionalized sensors, EZ-Link™ Amine-PEG_11_-Biotin (Thermo Fisher #26136) is coupled to the sensor, as described above. 1% ethanolamine is added and incubated for 30 min at room temperature. The sensor is placed in the GMR readout station, and 50 µL of streptavidin-coated magnetic nanoparticles (Miltenyi Biotec #130–048–101) is added and incubated for 15 min. The sensor is washed with 1 mL of PBS and then incubated for 21 days at 4°C under humid conditions by placing wet Kim wipes in a Petri dish with parafilm to create a seal. The stability evaluation is initiated by returning the assay to the GMR readout station for measurement. Next, the sensors are washed with 1 mL PBS, and streptavidin is blocked by incubating with 100 µL of 1 mM biotin in PBS. Stability is assessed by incubating the sensor array for 5 min each with 0.2, 1, and 5 M NaCl in water. The sensor is then washed briefly with water and sequentially incubated with PBS at 22, 4, and 50°C for 5 min each. For pH stability, the sensor is incubated in PBS adjusted to pH 3 with HCl. It is then incubated in PBS pH 7.2 and PBS adjusted to pH 9 and pH 13.5 with NaOH.

### Magnetic enzyme activity assay

The sensors are functionalized with a substrate (specific to the target enzyme) containing a biotin on one end and an amine group on the other. After overnight incubation with the substrate, the sensors are blocked for 30 min using 1% ethanolamine, followed by a 1 mL wash with PBS. The sensors are placed into the GMR readout station, and 50 µL of MNPs (Miltenyi Biotec #130–048–101) is added. The sensor resistance is measured continuously for 15 min. Then, a 1 mM solution of biotin in PBS is added for 15 min followed by a wash with 1 mL PBS. The enzyme solution is added to the sensors to initiate the magnetic enzyme activity assay, and the MR signal is measured.

### Nuclease assays

The sensors are functionalized with 9.56 ng of double-stranded DNA (5′– CCCCACTAGTAAAAAAAAAAAAAAAAAAAA–biotin–3′, Complementary: 5′– ACTAGTGGGGAAAAAAAAAAAAAAAAAAAA–NH_2_–3′), where the 3′end of one strand is derivatized with biotin and the 3′end of the other strand is derivatized with an amine group. The Bcu I recognition site (ACTAGT) is underlined. A double-stranded DNA sequence containing a scrambled Bcu I site (TACATG) was also synthesized as a control. The assay setup is described in the magnetic enzyme activity assay section above. To initiate the nuclease assay, 15 µL of the restriction enzyme Bcu I (Thermo Scientific #FD1254) is mixed with 45 µL of fast digest buffer (Thermo Scientific #FD1254) and added onto the sensors. Activity is monitored for 25 min at room temperature. The DNase I is set up under similar conditions, except for 3 units (3 µL) of DNase I solution in 97 µL of DNase I buffer (Thermo Scientific #EN0521). For the sequential Bcu I/DNase I assay, the sensors were functionalized using the Bcu I DNA substrate and the scrambled Bcu I sequence. 6.5 µL of Bcu I solution is combined with 43.5 µL of fast digest buffer for 15 min on the sensor. This is followed by a washing step with 1 mL of PBS before adding 5 units (5 µL) of DNase I in 45 µL of DNase I buffer for 10 min.

### Peptidase assays

The peptidase assay setup follows the protocol described in the nuclease assay, except the sensors are functionalized with a linker sequence consisting of biotin-PEG_36_-RQPVnWG-PEG_36_-NH_2_ or a scrambled version of the same peptide, biotin-PEG_36_-VWnRQGP-PEG_36_-NH_2_. The assay is initiated by adding 100 µL of 677 nM (20 μg/mL) human neutrophil elastase (Athens Research & Technology #16-14–051200) in PBS containing 0.01% Tween-20. To determine repeatability, three independent assays were performed using 100 μL of 500 nM human neutrophil elastase in PBS, 0.01% Tween-20, and the change in MR was recorded after 20 min of incubation. For the enzyme concentration curve assays, the enzyme was serially 2-fold diluted from 125 to 3.9 nM. For inhibition assays, the sensors are functionalized with the human neutrophil elastase substrate or its scrambled counterpart. The inhibitor, sivelestat sodium (VWR #89161–706), is introduced at 0, 4, or 20 μg/mL in a 30 µL PBS, 0.01% Tween-20 onto the sensors for 10 min. Subsequently, 677 nM of HNE is introduced into the inhibitor solution and the change in MR is recorded after 10 min.

### Cystic fibrosis sputum fluorescent assay

Sputum samples were collected from adult cystic fibrosis patients (>18  years) according to a UC San Diego institutional review board-approved protocol for human subject research (#160078) from the UC San Diego Adult Cystic Fibrosis Clinic during routine visits (Quinn et al., 2019). Samples were diluted 1:20 in PBS and stored at −20°C. Before use, samples are thawed, diluted 5-fold in PBS, and then mixed with an equal volume of 10 µM Ala-Ala-Pro-Val-7-amino-4-methylcoumarin (Alfa Aesar) in a black 384-well plate at a final volume of 30 µL such that the final dilution of sputum is 1 in 200. The reaction is incubated at 37°C for 2 h assays in a Synergy HTX microplate reader (BioTek), and readings are obtained in 47 s intervals at excitation and emission wavelengths of 360 and 460 nm, respectively. Enzyme velocity in relative fluorescent units per sec (RFU/s) is calculated using the highest slope recorded for 10 consecutive fluorescent readings, and the mean and standard deviation are determined from three technical replicates.

### Cystic fibrosis sputum magnetic assay

The cystic fibrosis sputum assay setup follows the protocol described above in the magnetic enzymatic activity assay section. The sensors are functionalized with the human neutrophil elastase substrate. Cystic fibrosis samples are prepared by diluting the frozen stocks 1:10 in PBS containing 0.01% Tween-20, then applying 50 µL of the diluted sample onto the sensors. The final dilution of sputum is 1 in 200.

### Statistical analysis and exclusion criteria

All data shown are the mean values with one standard deviation as error bars. Sensors that show a signal of more than 117 ppm or less than −117 ppm before MNP addition were excluded. In the hydrolase assays, the sensor was excluded if it did not have sufficient loading after MNP addition (3,300 ppm for human neutrophil elastase substrates and 470 ppm for restriction enzyme substrates). Statistical analysis (Pearson’s coefficient and Deming analysis) is done with custom-written code using NumPy (v1.18.5) and SciPy (v1.6.0) in Python (v3.8). The max negative velocity (-[Δ*MR*/*MR*_0_]/s) for the magnetic neutrophil elastase assays is calculated using LinearRegression from sklearn (v1.0).

## Results and discussion

### Surface chemistry

**FIGURE 2:**
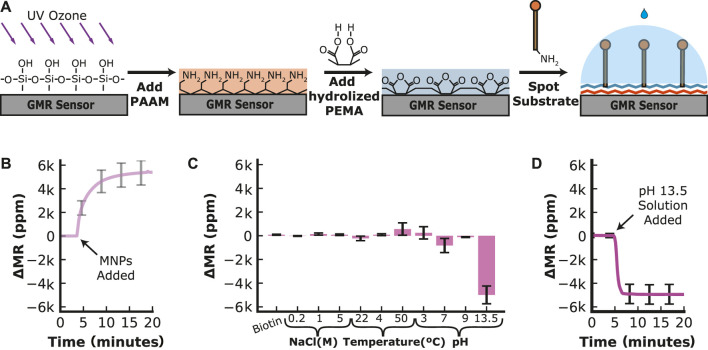
Sensor Functionalization and Stability of the Substrate. **(A)** The sensor surface is cleaned and activated by ultraviolet-ozone treatment before poly (allylamine) (PAAM) is added. The hydrolyzed poly (ethylene-alt-maleic anhydride) (PEMA) is added to create a layer of maleic groups. When the amine-containing substrate is spotted on the sensor surface, the maleic groups form a covalent bond with the amines on the substrate. **(B)** Time-dependent loading of streptavidin-MNP onto a sensor surface containing PEG_11_-biotin. **(C)** Change in magnetoresistance for a fully assembled PEG_11_-MNP complex that was stored for 21 days at 4°C and then sequentially incubated at various conditions. The change is recorded from one condition to the next. **(D)** Time-dependent decrease in signal in the presence of an extremely basic reagent (pH 13.5).

The efficacy of the GMR sensor platform hinges upon optimizing surface chemistry to ensure robustness and adaptability. This is crucial as instability of the immobilized substrate can affect the measurement of enzymatic activity and the sensor’s ability to be assembled and stored for use in a point-of-care setting. We previously showed that treating GMR sensors with polyallylamine (PAAM) followed by poly (ethylene-alt-maleic anhydride) (PEMA) yields a surface functionalized with maleic anhydride (Sveiven et al., 2023). Proteins that contain free amines, such as antibodies, can be covalently coupled to the surface for use in an immunoassay. Using this same surface chemistry approach, we coupled an amine-PEG_11_-biotin linker molecule to a GMR surface via an amine reaction with maleic anhydride (Figure 2A). Upon addition of streptavidin-coated MNPs, an average increase in resistance of 5,380 ppm was quantified on the 22 sensors containing amine-PEG_11_-biotin, while sensors that lacked this molecule had an average increase in resistance of 304 ppm, revealing that the biotin groups on the PEG linker are responsible for binding to the MNPs (Figure 2B). Following extensive washing with PBS, we obtained no noticeable reduction in signal, indicating that the MNPs are tightly bound to the sensor surface.

The functionalized sensor chip was stored at 4°C for 21 days to evaluate the long-term stability and then placed back in the GMR readout station. We then performed extensive washing steps using a range of buffer conditions to determine if the signal decreased. Little change (<5%) in resistance was quantified in excess biotin, revealing that PEG_11_-biotin was tightly bound to streptavidin-MNP and could not be competed off. Sequentially adding an increasing concentration of NaCl or changing the temperature from 22°C (room temperature) to 4°C did not alter the signal more than 5% of the saturated signal after MNP addition. When the temperature was changed from 4°C to 50°C, the signal increased by 9.6% of the saturated signal after MNP addition. The sensor was then washed with reagents buffered at various pH values. Changing the pH from neutral to pH 3 increased the signal by 4.9%, then decreased by 15.2% when the buffer was changed back to pH 7. Increasing the pH further to pH 9.0 has little effect on the signal (<5%). However, adding pH 13.5 buffer reduced the signal by 4,970 ppm, corresponding to a 92.5% reduction of the saturated signal after MNP addition (Figure 2C). By monitoring the real-time chemical release of MNPs under extreme alkaline conditions, we showed that the signal decreased rapidly within 3 min and then stabilized (Figure 2D). These data revealed that the sensor chip stored at 4°C for 21 days retained the PEG_11_ linker sequence and the streptavidin-coated MNPs and that this complex was only broken by treatment with a strong alkaline solution.

From these studies, it was unclear if the decrease in signal was due to the breakage of the amine-maleic anhydride bond or the streptavidin–biotin bond. To determine if the signal reduction was due to the release of the streptavidin–biotin, a reaction was set up whereby the linker-MNP complex was treated with a pH 13.5 solution, washed with PBS at neutral pH, and then incubated with fresh streptavidin-MNPs. The resistance signal decreased by nearly 75% upon treatment with pH 13.5 but then increased to 97% of its original signal upon adding fresh MNPs (Supplementary Figure S5). These studies reveal that the amine–maleic anhydride bond is stable in extreme alkaline conditions, but the biotin–streptavidin bond is broken, most likely due to the denaturation of streptavidin. The stability of the substrate-MNP complex on the sensor surface is compatible with the buffer conditions needed for hydrolase activity assays, as there are no documented human enzymes that require extreme alkaline conditions to be functional.

### Nuclease assays

**FIGURE 3:**
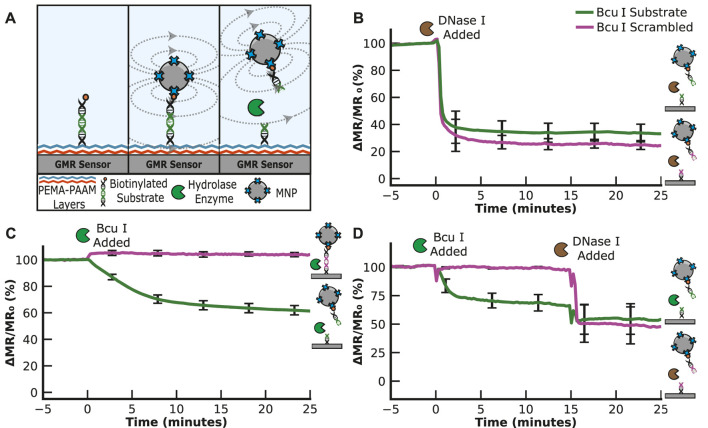
Nuclease Assay. **(A)** Graphical illustration of the nuclease assay. The signal decreases when the nuclease cleaves the substrate. **(B)** Hydrolysis of the ACTAGT (Bcu I substrate) and TACATG (Bcu I scrambled) sequences by DNase I results in a time-dependent decrease in the MR signal. **(C)** Hydrolysis of ACTAGT and not TACATG by the restriction enzyme, Bcu I. **(D)** Sequential addition of Bcu I and DNase I shows that Bcu I specifically cleaves the ACTAGT substrate, while DNase I cleaves the TACATG sequence.

We next evaluated the ability of hydrolytic enzymes to cleave a substrate sequence and, therefore, replaced the PEG_11_ linker with double-stranded DNA containing an amine group and biotin on each end (Figure 3A). We chose a sequence containing the restriction site for cleavage by Bcu I that corresponds to A*CTAGT, where * is the cleaved bond. This sequence is flanked by 4 bases on the 5′side and 20 bases on the 3′side and is coupled to the sensor surface as described for the PEG_11_ linker. Another DNA linker sequence was synthesized with a scrambled restriction site sequence, TACATG, which was expected to resist Bcu I cleavage. Upon cleavage of the DNA substrate, the bound MNPs are predicted to be released into solution, thereby reducing the magnetoresistance signal.

We found that the DNA sequences could be coupled to the sensor surface using the same chemical protocol optimized using the PEG_11_ linker. Following the wash steps, the signal was evaluated for 5 min to ensure stability. To determine if the two DNA sequences are accessible for nuclease cleavage, we added DNase I, a broad-spectrum nuclease enzyme that non-specifically cleaves the phosphodiester bonds in double-stranded DNA sequences. This enzyme rapidly cleaved both DNA sequences, decreasing the signal by 59.0% for the Bcu I substrate and 64.1% for the scrambled Bcu I substrate after only a 1-min incubation (Figure 3B). The signal stabilized, indicating that all available DNA linker sequences were hydrolyzed. These data confirm that the surface-tethered DNA sequence is accessible for cleavage by a nuclease.

To assess the specificity of the Bcu I substrate over the scrambled sequence, we added Bcu I to a sensor containing both sequences. After incubation for 25 min with the scrambled substrate, no reduction in signal was detected, indicating that this DNA sequence was not cleaved. However, in the adjacent sensors containing the Bcu I substrate, a time-dependent change in signal was detectable, with 22.9% reduction in signal within 5 min and an additional 21.2% reduction over the remaining 20 min (a total of 44.1% reduction in 25 min) (Figure 3C). We next set up an assay where Bcu I was incubated with the DNA sequences for 15 min, and then DNase I was added to the same sensor (Figure 3D). These studies showed that both DNA sequences are cleavable by DNase I, but only the ACTAGT sequence is a substrate for Bcu I.

Knowing that the DNA sequences can be cleaved by nucleases, we evaluated their stability in saliva, a biofluid of interest for use in point-of-care applications. Saliva contains numerous hydrolytic enzymes such as salivary amylase, peptidases, lysozyme, and lipase (Chojnowska et al., 2018; des Gachons and Breslin, 2016; Feng et al., 2019; Mennella et al., 2014). When exposed to saliva, the Bcu I substrate and scrambled DNA sequence had only a 1.3% and 1.6% reduction in signal after 10 min of incubation, respectively (Supplementary Figure S6). When compared to the reductions observed in the presence of Bcu I and DNase I, the signal change caused by saliva is statistically insignificant. These studies reveal that the DNA-MNP complex is stable in a complex biological sample containing numerous hydrolytic enzymes.

### Peptidase assays

After validating the MNP assay with a nuclease, we evaluated the assay format for a peptidase, an alternative hydrolase that cleaves peptide bonds instead of phosphodiester bonds. For these studies, we chose human neutrophil elastase (HNE) as the target enzyme as it has been established as a sputum biomarker for exacerbations associated with chronic obstructive pulmonary disease (COPD) and cystic fibrosis (CF). During neutrophil degranulation, elastase from granules is released and efficiently kills bacteria; however, the excess enzyme also damages lung tissue by degrading extracellular matrix proteins that are important for lung structure and elasticity (Mecham et al., 1997; Kafienah et al., 1998; Saetta et al., 2001; Kawabata et al., 2002). The amount of neutrophil elastase in sputum is directly proportional to the amount of activated neutrophils. We have detected neutrophil elastase activity in the sputum of patients with CF using fluorogenic substrates and revealed that patients with severe disease and more pathogenic bacteria have higher levels of elastase activity (Quinn et al., 2019). While numerous assays have been developed to quantify this enzyme, we were interested in designing a point-of-care peptidase assay using the MNP sensor system.

**FIGURE 4:**
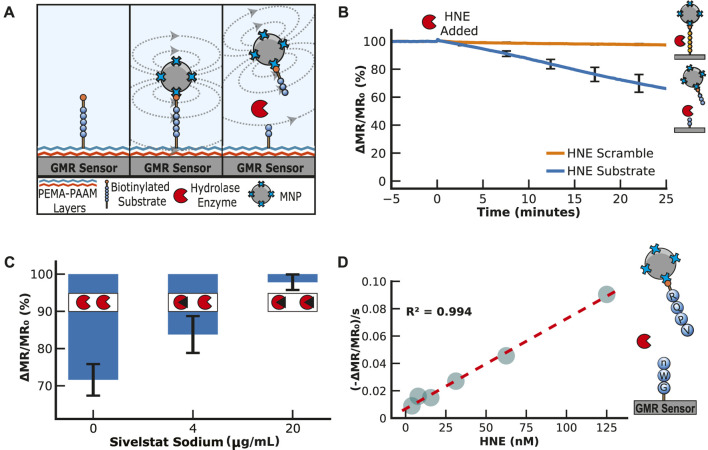
Human Neutrophil Elastase Assay. **(A)** Illustration of the neutrophil elastase assay. The peptide substrate is covalently attached to the sensor surface and then bound to streptavidin-MNPs via a biotin group on the peptide. This results in an increase in magnetoresistance. The addition of human neutrophil elastase cleaves the substrate, decreasing the signal. **(B)** An example of how the signal is loaded and then reduced by adding human neutrophil elastase. The signal is displayed as a percentage of the loading signal after the magnetic nanoparticle binding has saturated. **(C)** Human neutrophil elastase assays with inhibition by sivelestat sodium. All three assays have 20 μg/mL of neutrophil elastase, but the inhibitor concentration increases from 0 to 20 μg/mL. **(D)** Human neutrophil elastase titration serial diluting from 125 to 3.9 nM by a factor of 2 in PBS containing 0.01% Tween-20. The magnetic assay results are shown as the maximum velocity.

We first needed to find a peptide efficiently cleaved by HNE. In previous studies by our group, we incubated HNE with 124 different 14-mer peptides, each highly diversified in sequence. The enzyme cleaved 78 of these peptides, and a substrate specificity profile was generated using the most frequently found amino acids in each position surrounding the cleavage site (O’Donoghue et al., 2013). A consensus peptide sequence consisting of Arg-Gln-Pro-Val*Nle-Trp-Gly (RQPVnWG) was developed as a sequence cleaved by HNE, where * is the cleavage site and Nle (n) is norleucine, a non-natural amino acid. In parallel, we identified a scrambled peptide sequence, VWnRQGP, that contains the same seven amino acids but is not cleaved by HNE. These peptides were synthesized with a PEG_36_ linker on each end. On the N-terminal PEG_36_, an amine group was included to covalently attach to the sensor surface, while the C-terminal PEG_36_ contains a biotin group to bind MNPs (Figure 4A). Upon exposure of the sensor to a sample containing HNE, it was predicted that the RQPVnWG peptide would be cleaved between V and n, while the scrambled peptide would not be cleaved.

Both peptides were coupled to the sensor surface using the protocol outlined previously for PEG_11_ and DNA, and upon the addition of MNPs, a signal increased by ∼4,000 ppm, which confirmed the interaction between the biotin on the peptide with the streptavidin-coated MNPs. Following a wash step, the signal was monitored for 5 min to ensure stability, and then HNE was added. The peptide containing the substrate sequence was cleaved, releasing the MNPs into the solution in a time-dependent manner (Figure 4B). The signal from the scrambled peptide sequence remained unchanged in the assay. This confirmed that cleavage by HNE was specific for the RQPVnWG substrate and revealed that the enzyme does not cleave the streptavidin protein, which could non-specifically release the MNP due to the breakdown of the streptavidin-biotin interaction. Three independent assays were performed, and the coefficient of variation from all sensors after 20 min of incubation was calculated to be 11% (Supplementary Figure S7).

Next, we evaluated the ability of the assay to distinguish between active and inhibited HNE. Sivelestat sodium is a clinically approved HNE inhibitor for treating acute lung injury or acute respiratory distress syndrome (Pu et al., 2017). HNE was added to RQPVnWG sensors that contained either 4 or 20 μg/mL of sivelestat sodium, and the reactions were monitored for 10 min. The change in magnetoresistance was compared to the HNE digestion assay without the inhibitor. In the absence of sivelestat sodium, the signal decreased by 28.4%, while in the presence of 4 μg/mL, the signal decreased by 16.2%. At 5× higher inhibitor concentration, the signal decreases by only 2.2%, revealing that the enzyme was mostly inactivated under these conditions (Figure 4C). These data showed that the release rate of MNPs correlates with the amount of active enzyme in the assay. We next performed a serial dilution of HNE from 125 to 4 nM and calculated the change in MR signal per second at each concentration. From these studies, a linear concentration curve was calculated with an *R* ^2^ value of 0.994 (Figure 4D), confirming that the change in MR signal directly correlates with enzyme concentration.

**FIGURE 5:**
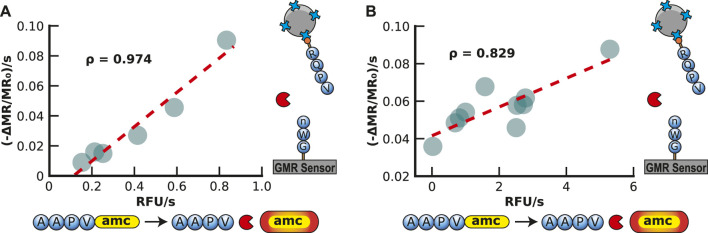
Validation of Magnetic Neutrophil Elastase Assay in Buffer and Sputum. **(A)** Validation of the magnetic human neutrophil elastase assay by comparison to a traditional AMC assay readout with a spectrometer. Each point represents a different concentration of human neutrophil elastase in buffer readout by the GMR readout station (shown in the y-axis) and the spectrometer (shown in the x-axis). The concentration ranges from 3.9 to 125 nM of human neutrophil elastase. **(B)** Validation of the magnetic human neutrophil elastase assay compared to a traditional amc assay readout with a spectrometer. Each point represents a different human sputum sample of patients with cystic fibrosis measured by the GMR station (shown in the y-axis) and the spectrometer (shown in the x-axis).

One of the most commonly used fluorogenic peptide substrates for monitoring HNE activity is Ala-Ala-Pro-Val-7-amino-4-methylcoumarin (AAPV-amc), where cleavage between V and amc results in an increase in fluorescence at 460 nm. To directly compare the peptide-MNP assay with a traditional fluorogenic peptide assay, the fluorogenic substrate was also assayed with 125 to 4 nM of HNE. The assay yielded an expected concentration-dependent increase in the reaction velocity. When comparing the velocities of both assays, a Pearson correlation coefficient of 0.974 was calculated, indicating a very strong positive correlation between the surface-based MNP release assay and the traditional solution-based fluorogenic assay (Figure 5A).

To evaluate the peptide-MNP assay using clinically relevant biofluids, we obtained sputum from 10 CF patients, diluted them 200-fold in assay buffer, and incubated it with both the peptide-MNP sensor and the fluorogenic substrate (Figure 5B). Each sputum sample contained sufficient HNE activity to release the MNPs, with the release rate ranging from 0.036 to 0.088 ppm/s. Compared with the fluorescent assay, the data was strongly correlative (Pearson correlation coefficient of 0.829). The velocity of MNP release by HNE in the sputum samples was then compared to the HNE concentration curve, and it was revealed that the amount of enzyme in each sample was between 8.8 and 24.6 μM (Supplementary Table S1). This concentration of HNE in these sputum is comparable to previous studies on CF sputum that used a colorimetric substrate to quantify HNE in CF sputum at a range of 0.47–18.5 μM (Rees et al., 1997; Dittrich et al., 2018). This study shows that the peptide-MNP assay applies to quantifying HNE in patient sputum samples.

### Potential use cases

The sputum samples used in this study were obtained from different patients; therefore, we could not perform a longitudinal study on how the HNE levels change when the patient is experiencing an exacerbation event. However, a potential application of this assay for patients with CF, COPD, or other lung diseases would be to monitor the HNE levels in sputum daily or weekly, thereby providing the healthcare team with data to monitor neutrophil levels in their lungs. The landscape of enzymes, their functions, and the impact of their dysfunction in disease states is vast. Table 1 lists a subset of these enzymes and the current methodologies used to measure their abundance. Nucleases and peptidases are activity-based markers for lung, inflammatory, and infectious diseases in addition to cancer. Many of these enzymes are currently assayed by ELISA, which cannot distinguish between active and inactive (inhibited enzymes or pro-enzymes). The standard fluorogenic methods for quantifying peptidase activity require a microplate reader and a trained technician. These methods can process many patient samples but are not amenable to point-of-care use. The magnetic enzymatic activity assay described here is designed for single-use in a point-of-care setting where the user (patient or healthcare provider) adds a biofluid sample to a pre-assembled substrate. The adaptability of the magnetic assay for other hydrolase substrates means that it can be readily modified to detect many different enzymes. The magnetic-based enzyme assay could greatly benefit the medical community as it seeks to diagnose patients and monitor their daily health.

**TABLE 1:** Potential applications of hydrolase activity assays.

Enzyme	Sample	Health condition	Use	Current assay methods	References
Human Neutrophil Elastase	Sputum	COPD, Cystic fibrosis, antibody-deficiency bronchiectasis	Exacerbation monitoring, Guide for antibiotic use	Activity-based immunoassay, Lateral flow device, Fluorogenic substrate-based kinetic assay, Mass spectrometry	Oriano et al. (2019), Thulborn et al. (2019), Chan et al. (2020), Voynow and Shinbashi (2021), Rofael et al. (2023)
Proteinase 3	Plasma, Sputum	α1-antitrypsin deficiency, Bronchiectasis	Exacerbation monitoring, Antitrypsin dosing guide	ELISA, Activity-based immunoassay	Newby et al. (2019)
Wound Peptidases	Wound Fluid	Chronic wounds	Early detection of non-healing wounds	Lateral flow device	Serena et al. (2021)
Gingipain	Saliva	Gingivitis	Detection of *Porphyromonas gingivalis*	Immunoassay, FRET substrate, Photoacoustic	Bikker et al. (2019), Hirai et al. (2020), Retout et al. (2023)
Human Nuclease	Tears	Dry eye disease	Disease monitoring	Gel electrophoresis, FRET	Sonawane et al. (2012)
Bacterial Nuclease	Urine	Urinary tract infection	Detection of urinary tract infections	FRET	Flenker et al. (2017), Machado et al. (2019), Qing et al. (2019)
DNase I	Serum	Stomach, colon, pancreas, breast, and oral cancer	Diagnosis	SRED, Microchip electrophoresis, ELISA, Immunochemical	Lauková et al. (2020), Balian and Hernandez (2021)
Prostate-specific Antigen	Serum	Prostate cancer, prostatitis	Screening, Risk stratification, Post-treatment monitoring	Lateral flow	Jung et al. (1999)
Trypsin	Blood	Acute pancreatitis, cystic fibrosis, and pancreatic cancer	Monitoring	ELISA, Colorimetry, Chemi-luminescence, Electrochemical, Photo-electrochemistry, and Fluorescence	Bao et al. (2021), Ping et al. (2021)
Matrix Metallo-proteinase-8	Gingival crevicular fluid	Periodontitis	Diagnosis	ELISA, Immunofluorimetry	Nazar Majeed et al. (2016)
Cathepsin S	Serum	Gastric cancer	Diagnosis and Prognosis	ELISA	Liu et al. (2016)
RNase 5	Plasma	Pancreatic cancer	Patient stratification	ELISA	Wang et al. (2018)
RNase A	Buffer	Many cancers	Diagnosis of malignant tumors and specific target for drug discovery	Electro-Chemiluminescence, Fluorescence, Chemiluminescence, Electrochemistry	Ni et al. (2019)
Pepsin	Saliva, Sputum	GORD, laryngopharyn-geal reflux, rhinitis, sinusitis, VFL	Possible biomarker	ELISA, HPLC	Stanforth et al. (2022)

## Concluding remarks

In this study, we report on a sensor platform for real-time hydrolase activity monitoring that improves upon some of the limitations of traditional methods. The field of bioengineering has long been accustomed to relying on optical assays to decipher enzymatic processes. However, the constraints of sample matrices, equipment complexity, and issues with autofluorescence and photobleaching have hindered their use. We introduce a novel sensor platform that quantifies hydrolase activity in a wash-free and highly adaptable assay. This adaptability is exemplified by incorporating either DNA or peptide substrates to facilitate the measurement of nucleases and peptidases, respectively. Moreover, the platform’s distinct attribute lies in its readiness for point-of-care diagnostics, a feature that traditional methods often struggle to accommodate. The inherent adaptability of our platform, coupled with its reliance on magnetic nanoparticles, enables the facile development of robust point-of-care assays. The resilience exhibited in complex biological matrices, such as saliva and sputum, further solidifies its applicability in scenarios requiring storage over extended periods.

In summary, the novel sensor platform, presented within the context of this study, emerges as a step forward in enzymatic activity analysis. This platform offers an adaptable and point-of-care capable solution by improving upon the limitations that have tethered traditional methods. With potential applications spanning diverse biomedical domains, this innovation paves the way for precision enzymatic activity analysis, promising to empower researchers and clinicians alike in exploring the complexities of enzymatic processes and their implications in health and disease.

## Data Availability

The original contributions presented in the study are included in the article/Supplementary Material, further inquiries can be directed to the corresponding authors.
