# Supplementary material for: A GMR enzymatic assay for quantifying nuclease and peptidase activity
Source: Front Bioeng Biotechnol. 2024 Mar 13;12:1363186. doi: 10.3389/fbioe.2024.1363186 (PMC10966768; doi:10.3389/fbioe.2024.1363186)
Supplement: Supplementary file 1 [file DataSheet1.pdf]

A GMR Enzymatic Assay for Quantifying Nuclease and Peptidase Activity

Supplementary Material

1 Supplementary Figures and Tables

1.1 Supplementary Figures

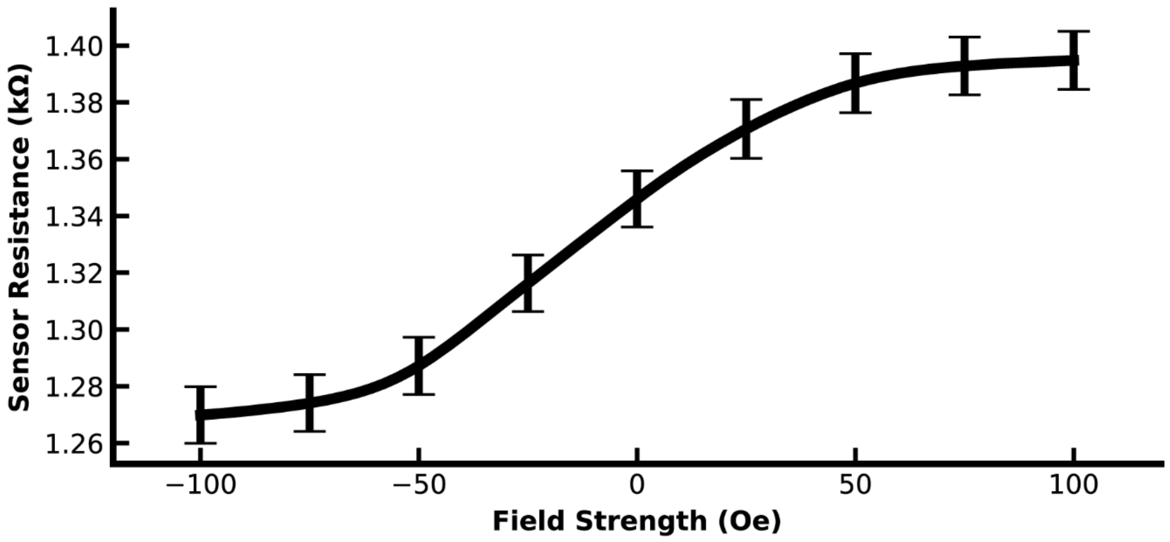

Supplemental Figure 1. GMR SV Transfer Curve. Measured resistance versus applied magnetic field for GMR SV sensor.

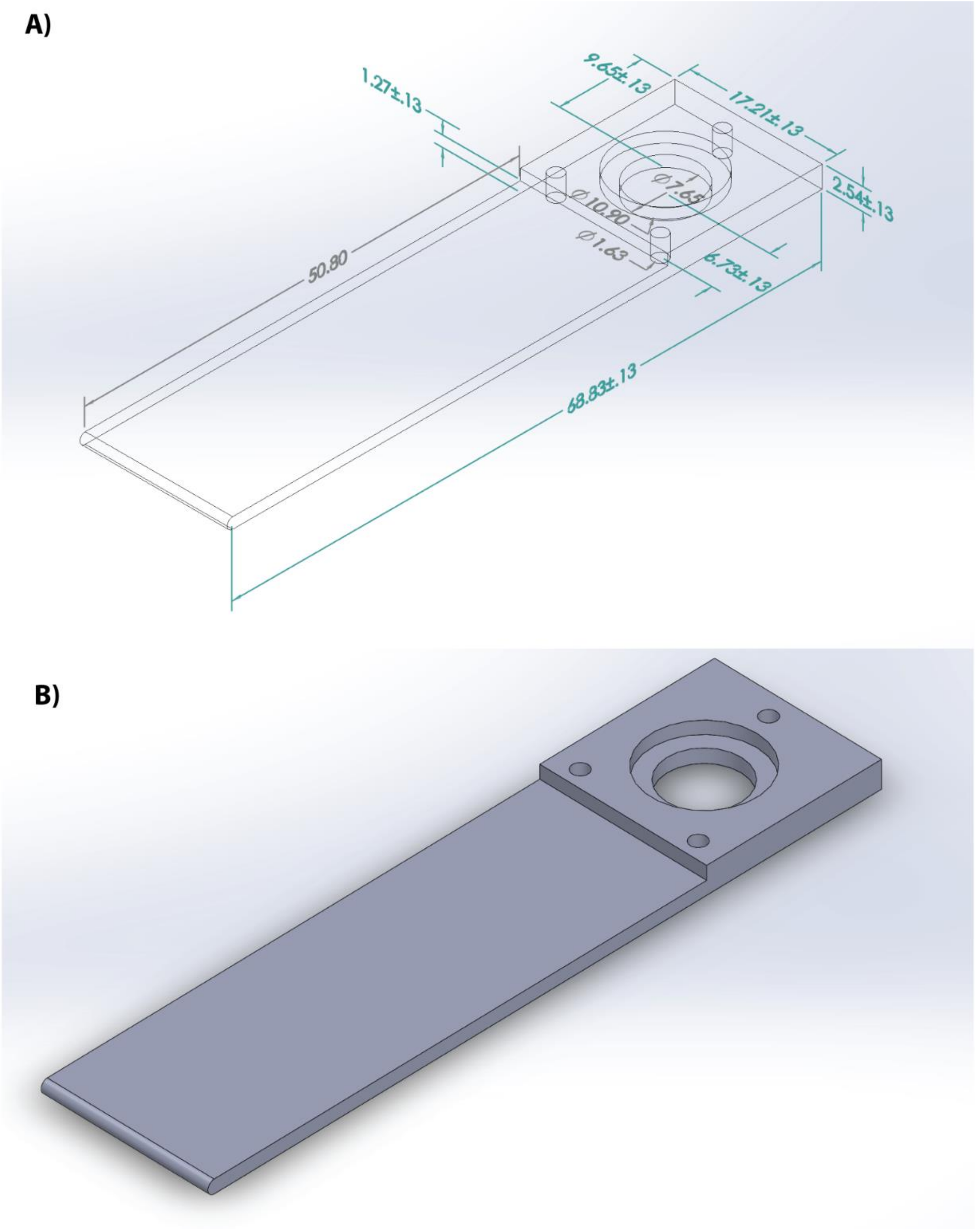

Supplemental Figure 2. Sensor Holder. A) Drawing of Teflon holder used to create a well over the GMR SV sensor array with dimensions in millimeters. **B)** 3D rendering of Teflon holder for GMR SV array.

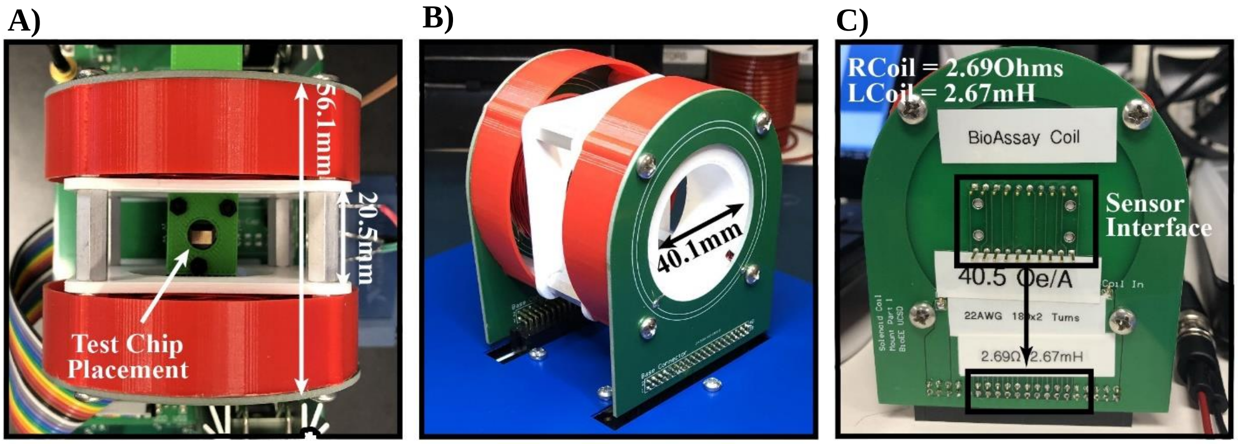

Supplemental Figure 3. GMR reader. A-C) Photographs of GMR SV reader station.

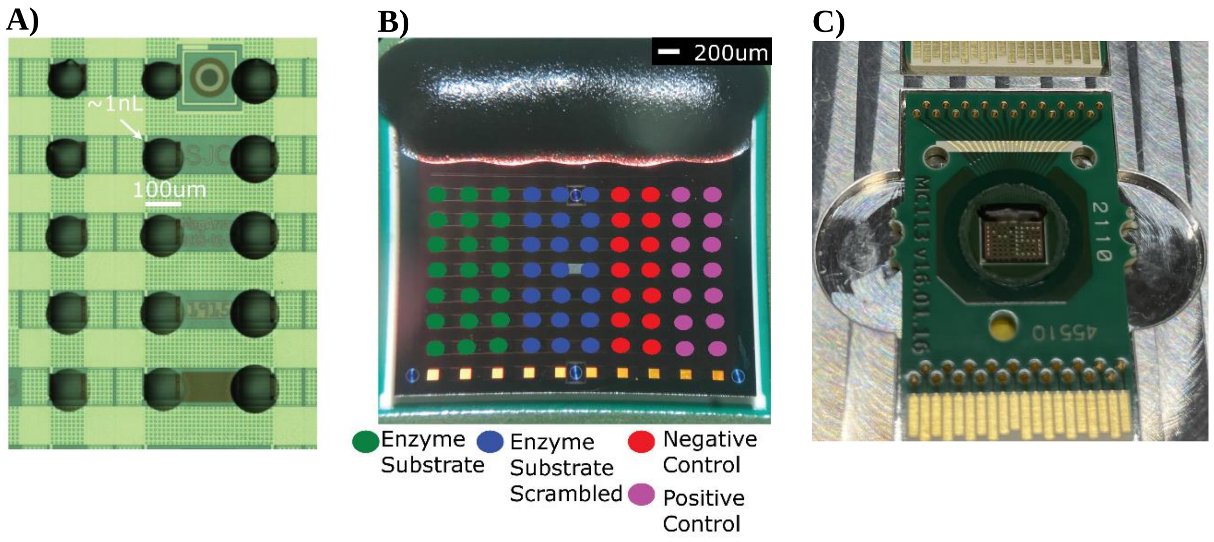

Supplemental Figure 4. **A**) Zoomed image of the GMR SV sensor array with sensors spotted with reagents. **B)** The GMR SV sensor array with 80 sensors arranged in an 8×10 matrix where each sensor is 120×120 μm^2 on a 280 μm pitch. Photograph shows an example mapping of the location of substrates and controls. **C)** Demonstration of precise liquid dispensing using an iTWO-300P automated spotter. The 34 droplets were individually spotted on sensors in a defined pattern. The example shows a spotting pattern with the first author's initials (MS).

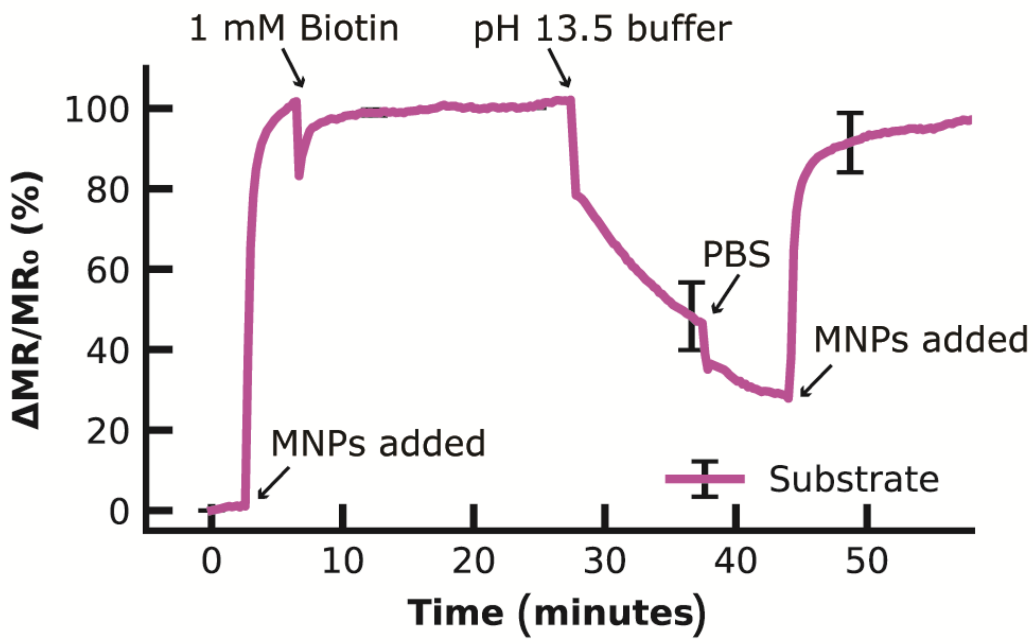

Supplemental Figure 5. Signal Reduction and Restoration. Demonstration of the loss of substrate signal due to the addition of pH 13.5 solution, but the restoration of that signal after washing and the addition of MNPs.

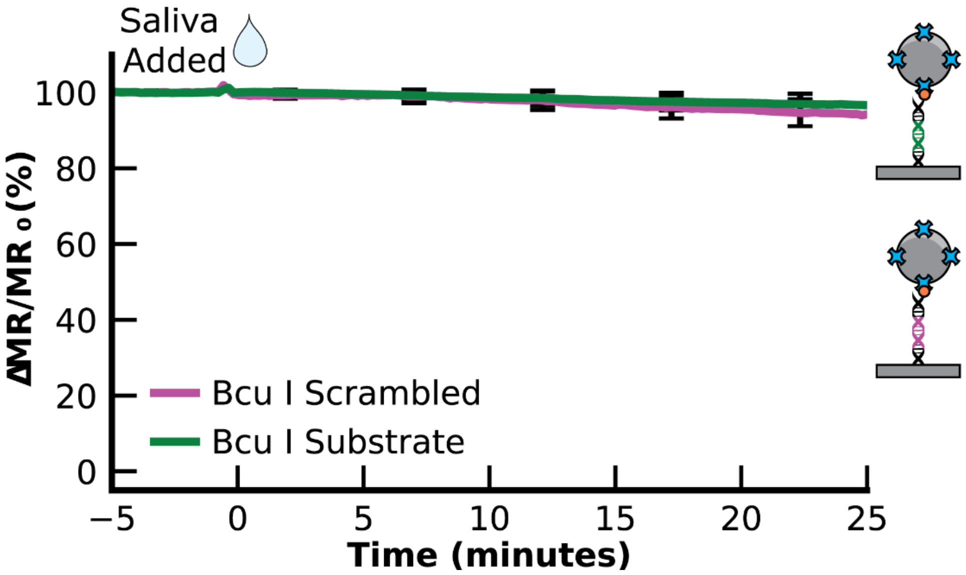

Supplemental Figure 6. Assay Stability in Saliva. Assays showing the stability of the substrates in saliva over 25 minutes of incubation. The sensors show less than a 5% reduction in signal for either the Bcu I substrate sensors or the Bcu I scrambled substrate sensors.

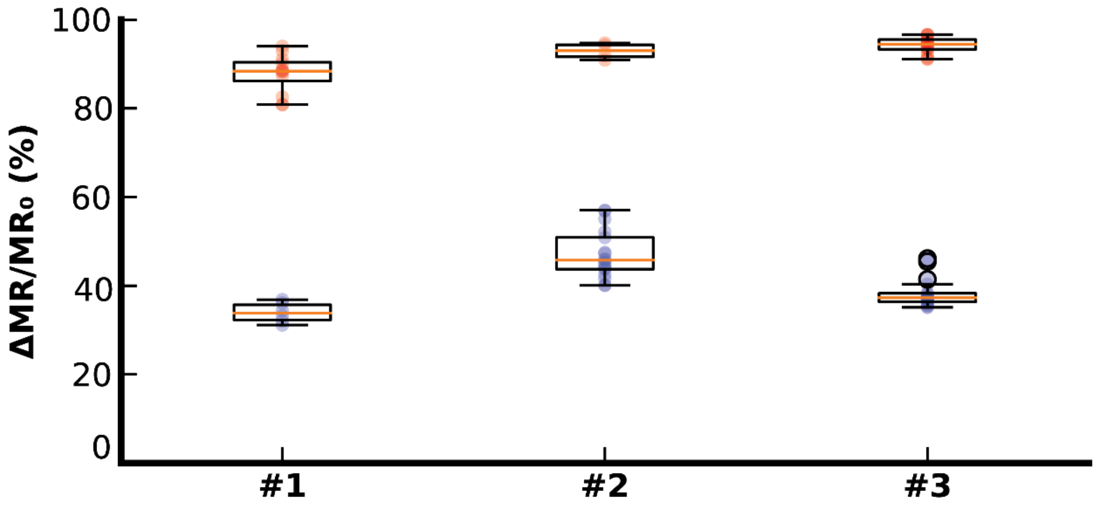

Supplemental Figure 7. Evaluation of Repeatability. Three independent assays were performed on replicate sensors to demonstrate repeatability. Sensors were functionalized with either the human neutrophil elastase substrate (blue) or the scrambled peptide (red). 500 nM of human neutrophil elastase was added to each sensor, and the change in signal was recorded after 20 minutes of incubation. The coefficient of variation (CV) from all sensors was calculated to be 11%.

1.2 Supplemental Tables

CF Sample	$(-\Delta MR/MR_0)/s$	HNE (nM) in assay	HNE (µM) in Sputum
1	0.046	59.32	11.86
2	0.051	67.22	13.44
3	0.058	78.06	15.61
4	0.058	77.47	15.49
5	0.088	122.89	24.58
6	0.062	83.37	16.67
7	0.054	71.87	14.37
8	0.048	63.25	12.65
9	0.036	44.13	8.83
10	0.068	92.56	18.51

Supplemental Table 1. CF Sample Concentrations Calculated from HNE Titration. The back-calculated concentration of active HNE in CF sputum (200-fold diluted) and the neat sputum sample.
